# Design and Realization of a Three Degrees of Freedom Displacement Measurement System Composed of Hall Sensors Based on Magnetic Field Fitting by an Elliptic Function

**DOI:** 10.3390/s150922530

**Published:** 2015-09-08

**Authors:** Bo Zhao, Lei Wang, Jiu-Bin Tan

**Affiliations:** Harbin Institute of Technology, D-403 Science Park, No. 2 Yikuang Street, Harbin 150080, China; E-Mails: hitzhaobo@hit.edu.cn (B.Z.); Jbtan@hit.edu.cn (J.-B.T.)

**Keywords:** Hall sensor, displacement measurement, magnetic field fitting, elliptic function

## Abstract

This paper presents the design and realization of a three degrees of freedom (DOFs) displacement measurement system composed of Hall sensors, which is built for the *XYθz* displacement measurement of the short stroke stage of the reticle stage of lithography. The measurement system consists of three pairs of permanent magnets mounted on the same plane on the short stroke stage along the *Y*, *Y*, *X* directions, and three single axis Hall sensors correspondingly mounted on the frame of the reticle stage. The emphasis is placed on the decoupling and magnetic field fitting of the three DOFs measurement system. The model of the measurement system is illustrated, and the *XY* positions and *θ_Z_* rotation of the short stroke stage can be obtained by decoupling the sensor outputs. A magnetic field fitting by an elliptic function-based compensation method is proposed. The practical field intensity of a permanent magnet at a certain plane height can be substituted for the output voltage of a Hall sensors, which can be expressed by the elliptic function through experimental data as the crucial issue to calculate the three DOFs displacement. Experimental results of the Hall sensor displacement measurement system are presented to validate the proposed three DOFs measurement system.

## 1. Introduction

Precision metrology plays an important role in on-machine measurement, positioning and manufacturing, such as machine tools, coordinate measuring machines and semiconductor manufacturing [1]. Particularly, the accuracy of these machines is significantly determined by the translational and rotational stages, so multi-DOF precision measurement of the stages is crucial. In recent years, abundant research on multi-DOF measurement systems for precision stages has been carried out. Aktakka developed a microactuation and sensing platform which can be used to provide precise physical reference for calibration of multi-degrees-of-freedom inertial sensors [2]. A six-DOF displacement measurement system using a diffraction grating as a cooperative target was proposed by Kim, combined with optical triangulation [3]. The Stewart parallel structure was applied in multi-DOF measurement as a MEMS sensor by Mura [4], and a sensitivity analysis of the configuration was carried on concerning geometrical characteristic and displacement amplitude [5]. Allred developed another Gough-Stewart platform-based measurement solution which integrates linear displacement sensors into a high capacity laminate bearing [6]. 

Hall magnetic field sensors feature contactless measurement, robustness, tolerance to harsh environments [7], simplicity and versatility [8], so they are widely applied in industrial control systems, precise instruments [9] and consumer electronic products, not only for direct measurement of magnetic fields [10], but also for non-direct measurements, such as speed or position [11,12,13], sometimes shape detection [14] or current measurement [15,16].

This paper presents a three DOFs displacement measurement system composed by three Hall sensors for precision positioning, which plays a crucial role while manufacturing or manipulating on the micro/nano level, where the key role of a precision positioning stage is to load, position and keep an object stable [17]. In this paper two translational DOFs and one rotational DOF of the stage are measured by three linear Hall sensors located in the same plane. A decoupling model of the measurement system is constructed, the stage positions can be obtained by the output of the sensors and the decoupling matrix. Then a magnetic field fitting method by an elliptic function is proposed to analytically express the magnet field line at a certain height, by which the practical field intensity of a permanent magnet can be substituted for the output voltage of the Hall sensors to solve the position of the stage. The decoupling and magnetic field fitting method is finally validated by experiments. 

## 2. Description of the Measurement System

**Figure 1:**
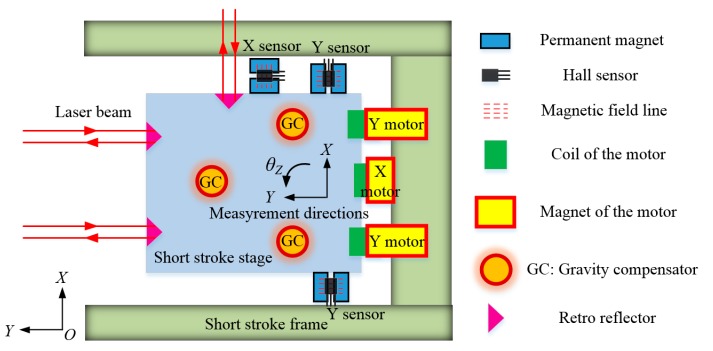
Schematic illustration of the proposed short stroke stage with 3D Hall sensors measurement system, in which the LS (long stroke) motors and the aerostatic guides are not included.

The *XYθ_Z_* displacements of the SS (short stroke) stage of the RS (reticle stage) of the lithography are measured by the cooperation of three Hall sensors in this paper. The schematic illustration of the SS stage is shown in Figure 1, in which the basic components of the SS stage are given. 

The SS stage is suspended by three gravity compensators (GCs) of cylindrical shape based on magnetic levitation. The GC is actuated by a voice coil motor along the *Z* axis, the GC rotors are connected with the SS stage, and the GC stators are fixed on the SS frame. Each GC can provide enough electromagnetic force along the *Z* axis for positioning. The radial gap between the magnet of the compensator and the coil is 2 mm, so besides *Z* displacement, the SS stage features *θ_X_* and *θ_Y_* displacements which can be also activated by the cooperation of the compensators. Meanwhile, the SS state can move virtually without friction in the *XYθ_Z_* DOFs. The *Zθ_X_θ_Y_* displacements can be measured by three Linear Variable Differential Transformers (LVDTs) mounted under the SS stage, by which the SS stage can be controlled to maintain an expected height. 

The *XYθ_Z_* DOFs of the SS stage can be achieved by the linear motors on the premise of suspended condition. In the *XOY* plane, the SS stage is actuated by three linear voice coil motors (LVCM) which are placed along the *Y*, *Y*, *X* directions, respectively, with the magnet of the motors fixed on the SS frame and the coil fixed on the SS stage. The center of gravity of the SS stage is not on the action line of the driving force of the motors, and each motor can generate both one force along its own axis and one torque along the *z* axis, so the motion in each of the three DOFs should be realized by the cooperation of the three motors. The movement along the *y* axis can be achieved by synchronous control of the two *Y* motors, with non-output of the *X* motor. The movement of the *X* motor can be achieved by the driving force along the *x* axis from the *X* motor, and a torque from two *Y* motors balanced with the torque from the *X* motor. Rotation along the *z* axis can be achieved by the reverse movement of the two *Y* motors which can simultaneously generate two opposite forces along the *y* axis and one torque along the *z* axis. The gap between the magnet and the coil of the LVCMs guarantees the movement of *XYθ_Z_* DOFs. The stroke of linear movement of the SS stage is 4 mm, that of rotation movement is 0.6°. The absolute position of the SS stage to the zero point of the lithography in the *XOY* plane is measured by three laser interferometers. Three pairs of permanent magnets used for *XYθ_Z_* displacements measurement are fixed on the SS stage along the *X* and *Y* directions, and the three corresponding Hall sensors are mounted on the SS frame above the permanent magnets along the direction of the equipotential line, so the movement of the SS stage relative to the SS frame perpendicular to the equipotential line in the *XOY* plane can be measured on the premise that the SS stage is held to the expected height by the three GCs, which is crucial for the relative motion control between the LS motion and SS motion. 

## 3. Model Analysis

### 3.1. Installation of the Hall Sensor

**Figure 2:**
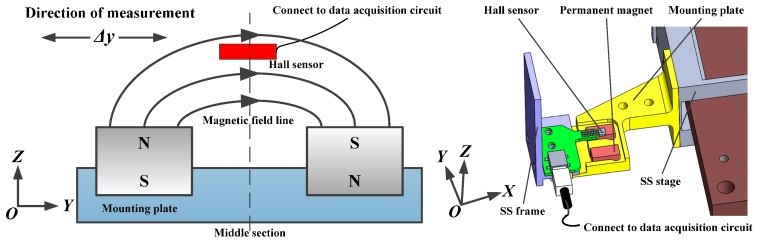
Installation of one Hall sensor and the permanent magnet.

The installation of each Hall sensor is shown in Figure 2. The output voltage of the Hall sensor is related to the magnetic flux density of the magnetic field lines passing through the sensor, which is the zero offset voltage in the middle section. Considering the motion range of the SS stage and the mounting space, the two pieces of permanent magnet are placed at a certain distance to construct an arc-shaped magnetic field above the upper surface of the magnet. The Hall sensor is fixed on the SS frame, and the mounting plate of the two pieces of permanent magnet is fixed on the SS stage. The permanent magnets are of the same size, with 12.7 mm length, and 6.4 mm width and height, and the installation dimensions of Hall sensor, including the terminal, are 43 mm × 25.5 mm × 6.5 mm. There is no mechanical interference during measuring with this installation method. 

**Figure 3:**
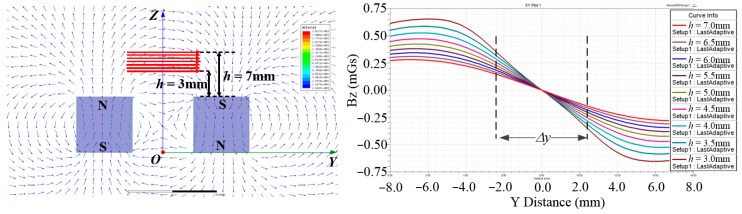
Simulation of the magnetic field, where *h* represents the height between the Hall sensor and the upper surface of the magnets.

The distribution of magnetic field above the upper surface of the magnet at different heights is simulated as shown in Figure 3, in which the curves of the relationship between magnetic flux density at a certain height and variation of position along *y* direction are given. The linear interval of the magnetic flux density can be used in displacement measuring. Although the nonlinear variation of magnetic flux density in response to the *y* position has disadvantages for measuring, a linear interval on the curves near the middle section exists, which is sufficient for measuring *y* displacements. 

### 3.2. 3-DOF Measuring Principle

**Figure 4:**
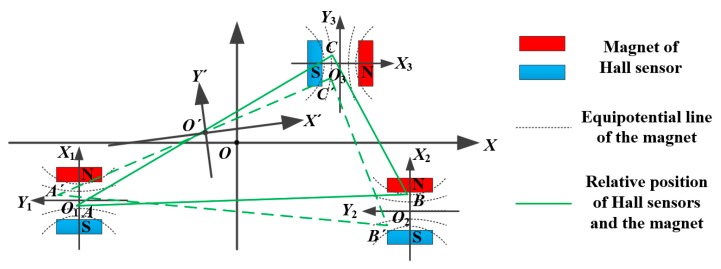
Measurement model of Hall sensors with three DOFs.

The measurement model of the Hall sensors with three DOFs is shown in Figure 4. The triangle *ABC* represents the arrangement of the three Hall sensors, which is invariable during measurements. In the Figure, the motion of the three pairs of magnets is replaced by the movement of the triangle *ABC* to *A'B´C´* for concise illustration. The coordinate system of the SS stage is denoted by *XOY*, and the sub-coordinate systems of the three Hall sensors are denoted by *X*_1_*O*_1_*Y*_1_, *X*_2_*O*_2_*Y*_2_ and *X*_3_*O*_3_*Y*_3_. In the sub-coordinate systems, the directions of measurement of the Hall sensors are along their own *X* axes which orient the *Y*, *Y*, *X* directions under the *XOY* coordinate system, respectively. The three pairs of magnets move with the SS stage, and the Hall sensors remain relatively stationary. 

The points *A*, *B* and *C* represent the original positions of the three Hall sensors relative to the magnet, and the points *A´*, *B´* and *C´* represent the current positions after a translational movement and rotation of the SS stage. Let (*x*_1_, *y*_1_), (*x*_2_, *y*_2_) and (*x*_3_, *y*_3_) be the coordinates of points *A*, *B* and *C* in the sub-coordinate systems, and (*X*_1_, *Y*_1_), (*X*_2_, *Y*_2_) and (*X*_3_, *Y*_3_) be the coordinates in the *XOY* coordinate system. Taking point *A* for example, which equipotential line the point *A* is standing on can be solved by the output of the Hall sensor, but the exact coordinates of *A* cannot be worked out. However, the shape of triangle *ABC* is constant, as it is related to the installation of the magnet. Once the equipotential line of each point is known, the exact coordinates of points *A*, *B* and *C* can be solved. 

Let $\vec{m}$ = (*x_m_*, *y_m_*) be the translation vector which represents the movement of the SS stage along the *X* and *Y* axes, and *θ* be the rotation angle about point *O*. The relationship between *ABC* and *A´B´C´* can be written as:

(1) $$\left(\begin{array}{c} x_{i}^{\prime }\\ {y^{\prime }}_{i} \end{array}\right)=\left[\begin{array}{cc} \cos\theta & -\sin\theta \\ \sin\theta & \cos\theta \end{array}\right]\left(\begin{array}{c} x_{i}\\ y_{i} \end{array}\right)+\left(\begin{array}{c} x_{m}\\ y_{m} \end{array}\right)\;(i=1,2,3)$$

Let (*X_O_*_1_, *Y_O_*_1_), (*X_O_*_2_, *Y_O_*_2_) and (*X_O_*_3_, *Y_O_*_3_) be the coordinates of the symmetric centers of the pairs of magnets in the *XOY* coordinate system, so the points *A*, *B* and *C* in the sub-coordinate systems can be transformed into the *XOY* coordinate system by:

(2) $${\left[\begin{array}{c} x_{1}\\ y_{1}\\ x_{2}\\ y_{2}\\ x_{3}\\ y_{3} \end{array}\right]}^{T}\left[\begin{array}{cccccc} \cos(\pi /2) & \sin(\pi /2) & 0 & 0 & 0 & 0\\ -\sin(\pi /2) & \cos(\pi /2) & 0 & 0 & 0 & 0\\ 0 & 0 & \cos(\pi /2) & \sin(\pi /2) & 0 & 0\\ 0 & 0 & -\sin(\pi /2) & \cos(\pi /2) & 0 & 0\\ 0 & 0 & 0 & 0 & \cos0 & \sin0\\ 0 & 0 & 0 & 0 & -\sin0 & \cos0 \end{array}\right]+{\left[\begin{array}{c} X_{O1}\\ Y_{O1}\\ X_{O2}\\ Y_{O2}\\ X_{O3}\\ Y_{O3} \end{array}\right]}^{T}={\left[\begin{array}{c} X_{1}\\ Y_{1}\\ X_{2}\\ Y_{2}\\ X_{3}\\ Y_{3} \end{array}\right]}^{T}$$

So if the value of points (*x_i_*, *y_i_*) and (*x_i_´*, *y_i_´*) can be obtained in response to the output of Hall sensors, the movement of the three DOFs of the SS stage in *XOY* plane can be given by:

(3) $$\{\begin{array}{l} x_{m}={x_{3}}^{\prime }-x_{3}\cos(2\arctan\alpha )+y_{3}\sin(2\arctan\alpha )\\ y_{m}=\frac{\Delta y_{1}(x_{2}^{2}+y_{2}^{2})+\Delta y_{2}(x_{1}^{2}+y_{1}^{2})-(x_{1}x_{2}+y_{1}y_{2})(\Delta y_{1}+\Delta y_{2})+2(x_{1}y_{2}-x_{2}y_{1})(y_{1}-y_{2})\tan\alpha }{{\left(x_{1}-x_{2}\right)}^{2}+{\left(y_{1}-y_{2}\right)}^{2}}\\ \theta =2\arctan\alpha \\ \tan\alpha =\frac{x_{1}-x_{2}+\sqrt{2[\Delta y_{1}\Delta y_{2}-x_{1}x_{2}-(\Delta y_{1}-\Delta y_{2})(y_{1}-y_{2})]-\Delta y_{1}^{2}-\Delta y_{2}^{2}+x_{1}^{2}+x_{2}^{2}}}{\left(\Delta y_{1}-\Delta y_{2})+2(y_{1}-y_{2}\right)} \end{array}$$

where *Δy_i_* = *y_i_´−* *y_i_* (*I* = 1, 2). In practical applications, the value of *θ* is less than 2º, and the line connecting the center of two Hall sensors of *Y* direction is not parallel to the *X* axis, so the denominator in Equation (3) is not zero, and the equation has a solution. 

It can be found in Figure 4 that the equipotential line of the magnet is a series of nonlinear curves, that is, the displacement of magnet in the measuring direction of Hall sensor cannot be directly calculated by the output of Hall sensors unless the distribution of the magnetic field line is known, so the key point of measuring the three DOFs is working out the relationship between the output of the Hall sensors and the relative position of the sensors in the magnetic field. 

## 4. Magnetic Field Fitting by an Elliptic Function

The Hall sensors are transducers which change the output voltage in response to a magnetic field while measuring. Therefore, Equation (4) representing the relationship between the output of the Hall sensors and the relative positions of the sensors in the magnetic field is crucial for displacement measuring, that is, once the output voltage of the Hall sensor is known, the equipotential line on which the Hall sensor is located can be found:

(4) U=F(x,y)

In this paper, a magnetic field fitting method is proposed to get the position coordinates from the output voltage of the Hall sensors. The equipotential lines of the magnet are approximated to conics, so the magnetic field could be fitted by conic curves. The possible types of conic curves are given in Table 1, which is used for solving the coordinates of the points *ABC* through the outputs voltage of the Hall sensors. 

**Table 1:** Possible types of conic curves.

Type	Expression
Elliptic	${\left(\frac{x_{n}-f_{n1}(U)}{f_{n2}(U)}\right)}^{2}+{\left(\frac{y_{n}-f_{n3}(U)}{f_{n4}(U)}\right)}^{2}=1,\;n=1,2,3;$
Parabolic	$x_{n}=f_{n1}(U){\left[y-f_{n2}(U)\right]}^{2}+f_{n3}(U),\;n=1,2,3;$
Hyperbolic	${\left(\frac{x_{n}-f_{n1}(U)}{f_{n2}(U)}\right)}^{2}-{\left(\frac{y_{n}-f_{n3}(U)}{f_{n4}(U)}\right)}^{2}=1,\;n=1,2,3;$

### 4.1. Data Collecting System

**Figure 5:**
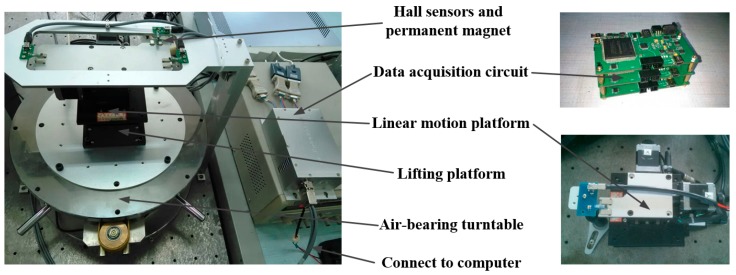
Data collection system with four DOFs.

A four DOFs magnetic field intensity collecting system is built for magnetic field fitting, which is composed of an air-bearing turntable, a lifting platform and a linear motion platform with two DOFs as shown in Figure 5. The linear motion platform is mounted on the upper surface of the lifting platform which is supported by the air-bearing turntable. The *θ_Z_* displacement can be achieved by the air-bearing turntable which features high rotation positioning accuracy of 1″. The resolution of *X* and *Y* displacements is 0.1 μm. The three pairs of magnets are fixed on the upper surface of the linear motion platform, and the corresponding Hall sensors are fixed on a gantry stack. During the data collection procedure, the Hall sensors remain stationary while the magnets move with the platforms, which is consistent with the real application. 

**Figure 6:**
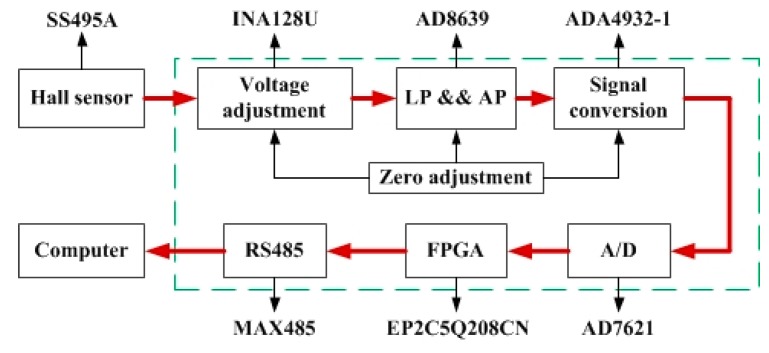
Structure of data acquisition circuit.

The data acquisition circuits are developed to improve the resolution and stability of the Hall sensors. According to the output signal and the magnetic field of the magnets, each Hall sensor features its own data acquisition circuit, of which the structure is shown in Figure 6. SS495A is the Hall sensor. The voltage adjust module is built by INA128U as a preamplifier which can satisfy the need of resolution and Signal to Noise Ratio (SNR). The Sallen-Key second order low pass filter is built with an AD8639 to acquire the rapidly decreasing signal at the cut off frequency. The output voltage of the low pass filter is converted to a differential signal by an ADA4932-1. The Field-Programmable Gate Array (FPGA) is the main controller which controls data acquisition and transmits digital signals to computer through RS485.

**Figure 7:**
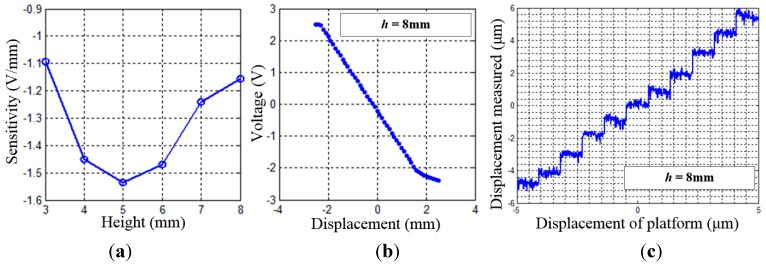
Performance of single Hall sensor: (**a**) Sensitivity on different height; (**b**) Output voltage along measurement direction on the plane of *h* = 0.8 mm; (**c**) Resolution of the Hall sensor along the measurement direction.

The performance of a single Hall sensor is tested by the data acquisition system. The sensitivity of the Hall sensor at different heights is examined as shown in Figure 7a; the height between the Hall sensor and the magnets is regulated by the lifting platform, and the displacement along the measurement direction is generated by the linear motion platform. The stroke of the platform in each plane is from 2.5 mm to 2.5 mm, with a 100 μm step size. It can be found that the sensitivity of Hall sensor is not monotonic versus *h*, and the optimal sensitivity occurred when *h* = 5 mm, where the output of the Hall sensor varies 1.53 V while the motion platform moves 1 mm. When *h* = 8 mm, the sensitivity of the Hall sensor is 1.15 V/mm, which is lower, but still sufficient for the displacement measurement. However, the installation space is limited by the frocks of Hall sensor and other restrictions from the motors and cables, so the *h* is determined to be 8 mm.

Considering the installation, voltage sampling range of the A/D chip and measurement range of the Hall sensor, the height between the Hall sensor and the magnets is selected to be 8 mm. The resolution of the Hall sensor in this case is 1 μm, as shown in Figure 7c. 

### 4.2. Magnetic Field Fitting by Elliptic Function 

**Figure 8:**
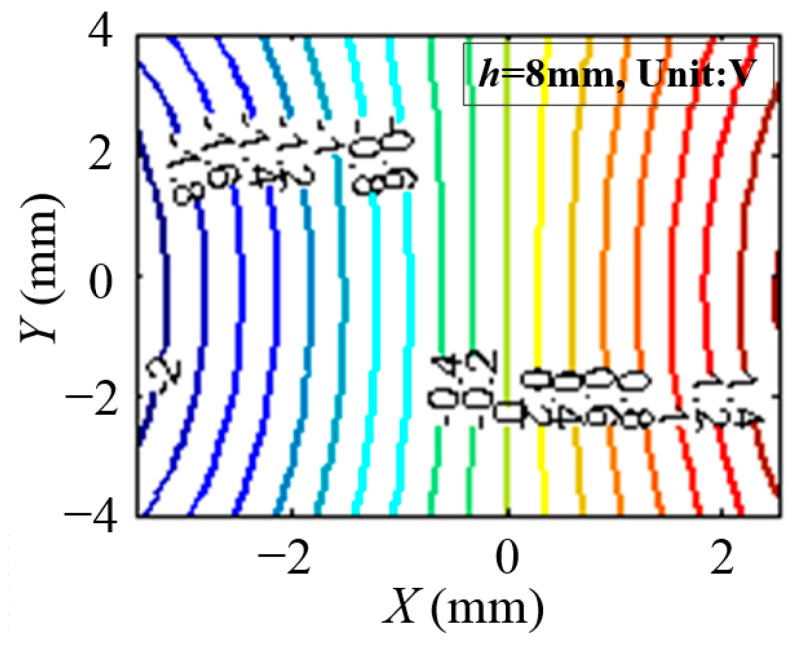
Equipotential line of the magnetic field represented by different colors.

The magnetic field intensity can be substituted for the output voltage of the Hall sensor. Therefore, the data of output voltage on the plane of *h* = 0.8 mm in the area of {(x, y)||x| ≤ 3.0 mm, |y| ≤ 4.0 mm} is collected, in which the measurement direction of the Hall sensor is along the *X* axis. Taking the installation errors and machining errors of the system into account, the data collection range is larger than the measurement range of the sensors. The interval of the data is 200 μm along the *X* axis, and 400 μm along the *Y* axis, so the voltage result data can make up to a 31 × 21 matrix. The equipotential line of the magnetic field is drawn by the data of output voltage as shown in Figure 8. The intensity of the magnetic field is indicated by the absolute value of the voltage, and the different colors represent the output voltage of the Hall sensor, which is related to the measurement direction. The voltage interval is 0.2 V. Then the equipotential line is fitted by the three types of function as listed in Table 1, respectively, to find out the most appropriate one to describe the real distribution. 

As shown in Figure 8, every equipotential line is considered to be composed of a series of discrete points of the voltage matrix, so taking the voltage of *U* = 0.4 V for example, the curve can be fitted by a parabolic equation (Equation (5)) with a certain value of *f*_11_(*U*), *f*_12_(*U*) and *f*_13_(*U*). The parameter *f*_11_(*U*) is one element of *f_n_*_1_(*U*) in the parabolic type as listed in Table 1, as well as the parameter *f*_12_(*U*) and *f*_13_(*U*).

(5) $$x=f_{11}(U){\left[y-f_{12}(U)\right]}^{2}+f_{13}(U)$$

**Figure 9:**
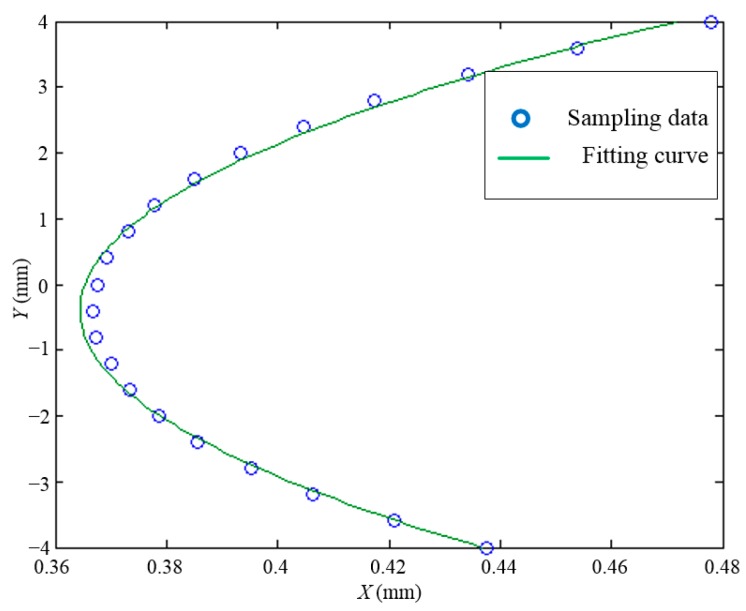
Equipotential line of *U* = 0.4 V fitted by Equation (5).

It can be found in Figure 9 that not all points lie on the curve, so the degree of fitting is evaluated by the variance of the points. After all equipotential lines are fitted by a parabolic function, the equipotential lines in Figure 8 are also fitted by an elliptic function and hyperbolic function. The variance of every equipotential line is listed in Table 2. It can be concluded that the equipotential lines fitted by the elliptic function can achieve the optimal result.

**Table 2:** Variance of fitting data by different models.

Voltage of Equipotential Line	Variance of Fitting Data			Voltage of Equipotential Line	Variance of Fitting Data		
	Parabola	Ellipse	Hyperbola		Parabola	Ellipse	Hyperbola
−2.0	1.36 × 10^−5^	1.97 × 10^−^^6^	1.37 × 10^−5^	0.0	1.66 × 10^−^^7^	1.65 × 10^−^^7^	1.72 × 10^−^^6^
−1.8	1.29 × 10^−^^4^	9.33 × 10^−^^6^	1.30 × 10^−^^4^	0.2	4.97 × 10^−^^6^	3.21 × 10^−^^6^	4.98 × 10^−^^6^
−1.6	1.47 × 10^−^^4^	1.03 × 10^−^^6^	1.48 × 10^−^^4^	0.4	4.54 × 10^−^^6^	1.71 × 10^−^^7^	4.58 × 10^−^^6^
−1.4	9.28 × 10^−^^5^	1.06 × 10^−^^6^	9.33 × 10^−5^	0.6	1.02 × 10^−5^	1.45 × 10^−^^7^	1.03 × 10^−5^
−1.2	5.90 × 10^−^^5^	8.40 × 10^−^^6^	5.93 × 10^−5^	0.8	2.43 × 10^−5^	5.40 × 10^−^^7^	2.45 × 10^−5^
−1.0	3.71 × 10^−5^	3.75 × 10^−^^7^	3.73 × 10^−5^	1.0	3.94 × 10^−5^	5.03 × 10^−^^7^	3.96 × 10^−5^
−0.8	1.90 × 10^−5^	2.58 × 10^−^^7^	1.91 × 10^−5^	1.2	6.06 × 10^−5^	4.74 × 10^−^^7^	6.10 × 10^−5^
−0.6	1.01 × 10^−5^	1.96 × 10^−^^7^	1.01 × 10^−5^	1.4	2.00 × 10^−5^	1.91 × 10^−^^7^	2.02 × 10^−5^
−0.4	4.82 × 10^−^^6^	1.21 × 10^−^^7^	4.84 × 10^−^^6^	1.6	1.02 × 10^−^^7^	1.29 × 10^−^^9^	1.02 × 10^−^^7^
−0.2	1.20 × 10^−^^6^	2.05 × 10^−^^7^	1.20 × 10^−^^6^	--	--	--	--
Variance of mean	3.57 × 10^−^^5^	6.52 × 10^−^^7^	3.60 × 10^−^^5^	--	--	--	--

Therefore it is decided to fit the magnetic field distribution with an elliptic equation (Equation (6)), and the focus is placed on the solution of parameters *f_n_*_1_(*U*), *f_n_*_2_(*U*), *f_n_*_3_(*U*) and *f_n_*_4_(*U*), which is also solved by a curve fitting method:

(6) $${\left(\frac{x_{n}-f_{n1}(U)}{f_{n2}(U)}\right)}^{2}+{\left(\frac{y_{n}-f_{n3}(U)}{f_{n4}(U)}\right)}^{2}=1$$

**Figure 10:**
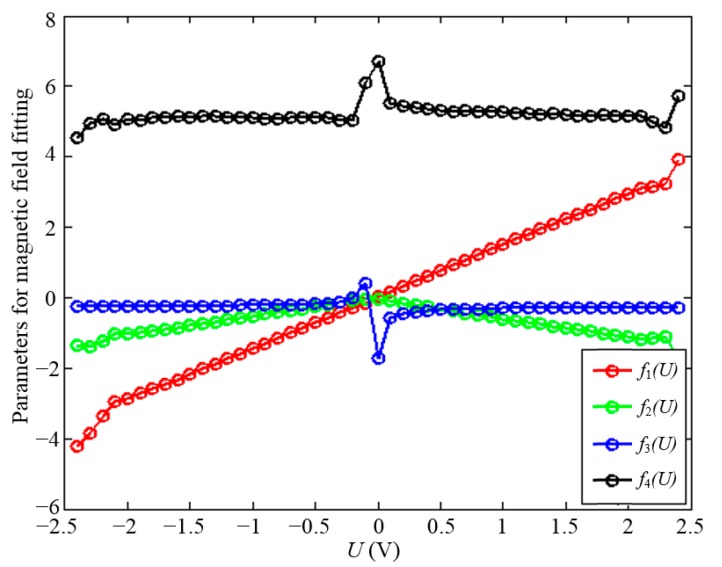
Parameters of the elliptic function. Each curve represents one parameter in Equation (6), which should be given by analytic expression.

To decrease the fitting error of the parameters, the voltage interval of the equipotential line is reduced to 0.1 V. The parameters of every equipotential line are solved as shown in Figure 10, each parameter is composed of a series of voltage, which can also be fitted by the proper function. The characteristics of each parameter are as follows:

1. The curve represented by *f_n_*_1_(*U*) is approximately linear if the outliers on both ends are ignored, and can be fitted by a linear function given as *f_n_*_1_(*U*) = *a∙U* + *b*;
2. The curve represented by *f_n_*_2_(*U*) is symmetric to the vertical axis *U* = 0 V, and is also approximately linear on one side, so it can be fitted by the absolute form of a linear function as *f_n_*_2_(*U*) = −|*c∙U* + *d|*. Taking the quadratic form of parameter *f_n_*_2_(*U*) in Equation (6) into account, the term *f_n_*_2_(*U*) could also be expressed by *f_n_*_2_(*U*) = *c∙U* + *d*;
3. The curve represented by *f_n_*_3_(*U*) is similar to an inversely proportional function, which can be written as *f_n_*_3_(*U*) = *p*/(*U* + *q*) + *m*;
4. The curve represented by *f_n_*_4_(*U*) is composed of irregular points. However, the effect of *f_n_*_4_(*U*) on the elliptic function mainly concentrates on the degree of convergence of the curvature radius of the equipotential lines, which influences more the points far from the origin but less near the origin, so that the *f_n_*_4_(*U*) is considered to be constant in the elliptic function.

Therefore, the four parameters in the elliptic function can be given by:

(7) $$\{\begin{array}{l} f_{1}(U)=1.479U+0.027\\ f_{2}(U)=0.565U+0.025\\ f_{3}(U)=-0.042/(U+0.031)-0.2661\\ f_{4}(U)=5.20 \end{array}$$

**Figure 11:**
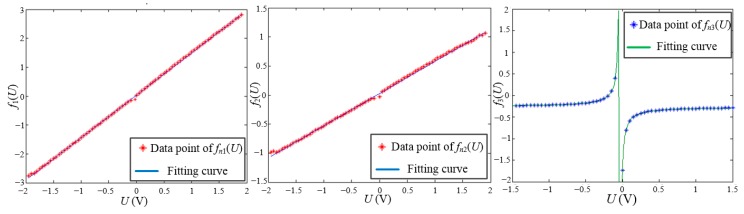
Fitting performance of the parameters *f_n_*_1_(*U*), *f_n_*_2_(*U*) and *f_n_*_3_(*U*).

The fitting performance of the parameters *f_n_*_1_(*U*), *f_n_*_2_(*U*) and *f_n_*_3_(*U*) is shown in Figure 11. The parameters of the elliptic function could be well fitted by Equation (7). So the elliptic function is given by:

(8) $${\left(\frac{x-1.479U-0.027}{0.565U+0.025}\right)}^{2}+{\left(\frac{y+0.042/(U+0.031)+0.2661}{5.20}\right)}^{2}=1$$

**Figure 12:**
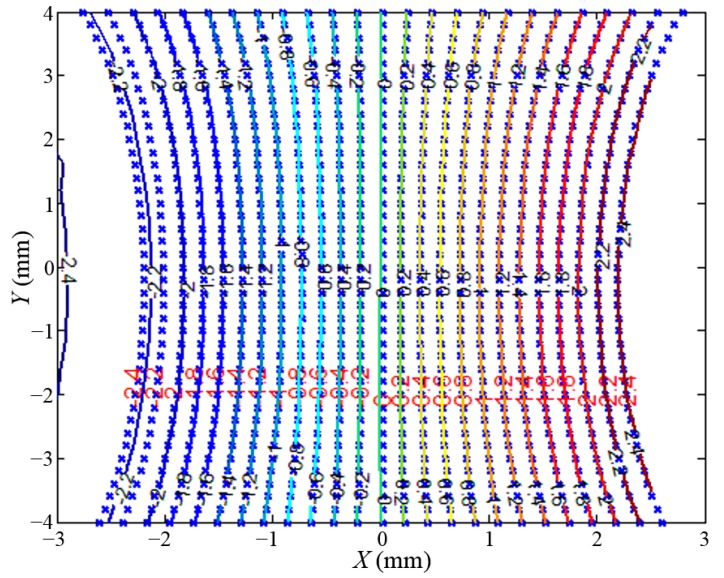
Fitting performance of the parameters *f_n_*_1_(*U*), *f_n_*_2_(*U*) and *f_n_*_3_(*U*).

Figure 12 shows the equipotential lines of the magnetic field fitted by Equation (8). The blue points represent the output voltage collected by the data collection system, and the curves with different colors represent the equipotential lines drawn by Equation (8). In the range of |*x*| ≤ 2.0 mm, corresponding to a voltage from −1.8 V to 1.8 V, the discrete points from the experiment can be substituted by curves. Considering measurement range requirement of the Hall sensor is 4 mm, it can be theoretically concluded that the magnetic field fitting method is applicable. 

## 5. Performance

**Figure 13:**
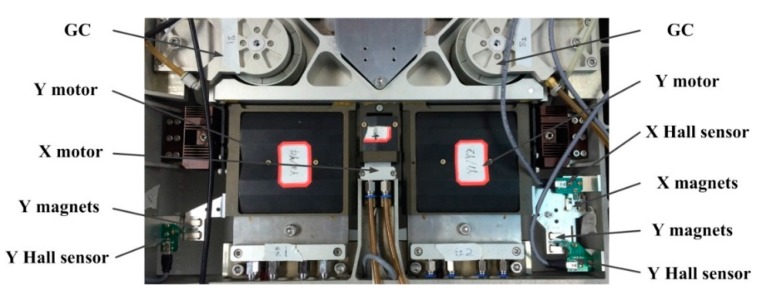
Three DOFs displacement measurement system installed on reticle stage of the lithography.

The performance of the three DOFs displacement measurement system is tested in a real application as shown in Figure 13. The three Hall sensors are installed along the *Y*, *Y*, *X* directions, which are the same as in the data collection system. The relative height of the Hall sensor and magnets is maintained the same as in the data collection system by the three GCs. The SS stage in the experiment corresponds to a closed-loop system which is driven by the three motors and measured by laser interferometers in the *XOY* plane, with the LS stage in a stationary state, so the data from the laser interferometers can be used as the reference of the Hall sensors. Before moving the SS stage, the initial Hall voltage *U_n_* of each sensor is recorded. Then the SS stage is actuated to move along the *X* and *Y* directions, respectively, to obtain the displacement sensitivity *k_n_* of the *X* Hall sensor and *Y* Hall sensor. 

**Figure 14:**
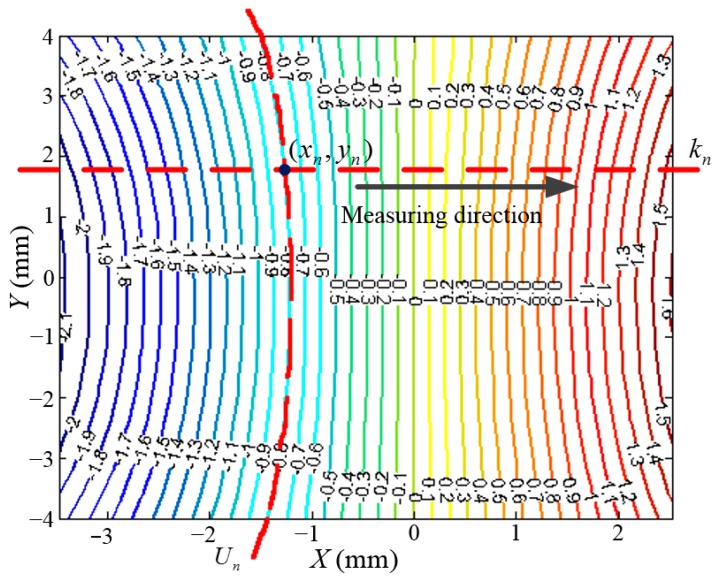
Calibration of the initial position of *X* Hall sensor.

Taking the *X* Hall sensor for example, as shown in Figure 14, the equipotential lines of the magnetic field are replaced by a voltage with a step of 0.1 V. The Hall sensor is located on the red elliptic curve if *U_n_* = −0.8 V. Then the SS stage moves along the *X* direction, and the displacement sensitivity *k_n_* can be calculated by the *X* displacement measured by laser interferometers and the variation of the Hall voltage, which is the red line in Figure 14. The magnetic field is fitted in the data collection system, that is, the corresponding Hall voltage of the magnetic field is known. *U_n_* is the initial voltage of the *X* Hall sensor, *k_n_* is the displacement sensitivity along the measuring direction. The intersection of the two red curves is the initial position of the *X* Hall sensor, so the initial position of *X* Hall sensor (*x_n_*, *y_n_*) is located on the intersection of the two red curves. 

**Figure 15:**
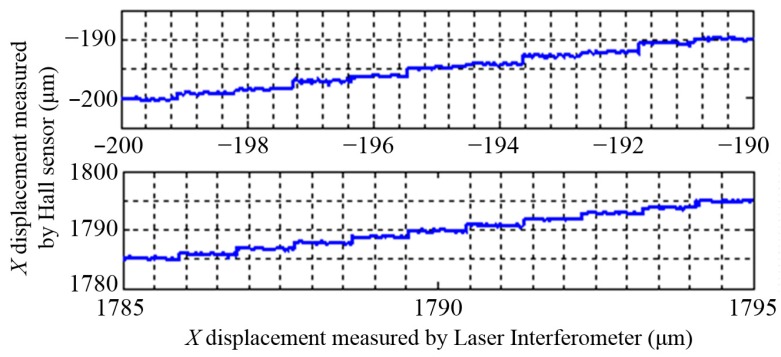
The resolution of the Hall sensors in *X* direction.

**Figure 16:**
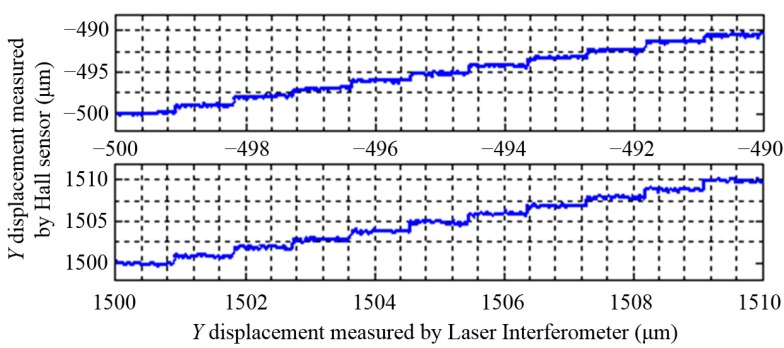
The resolution of the Hall sensors in *Y* direction.

**Figure 17:**
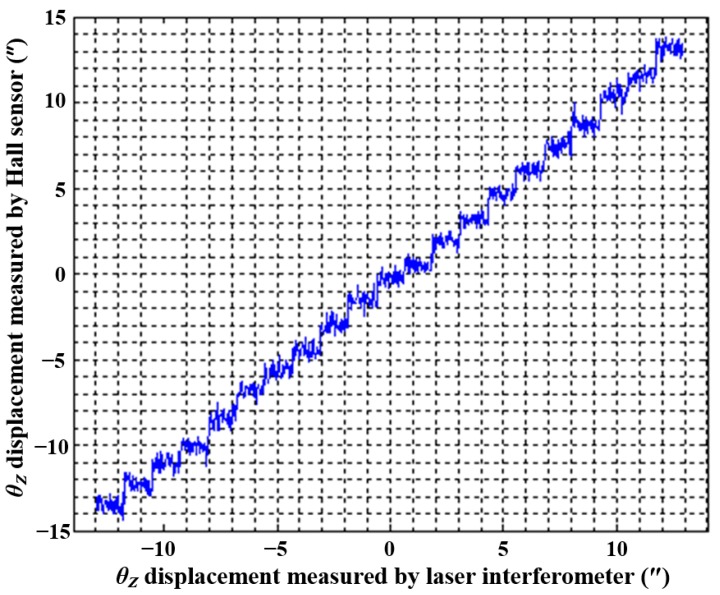
The resolution of the Hall sensors in *θ_Z_* direction.

The resolutions of the three DOFs displacement measurement system are shown in Figure 15, Figure 16 and Figure 17, the resolutions of *X* and *Y* displacements is 1 μm, and the resolution of the *θ_Z_* displacement is 1.3″. 

**Figure 18:**
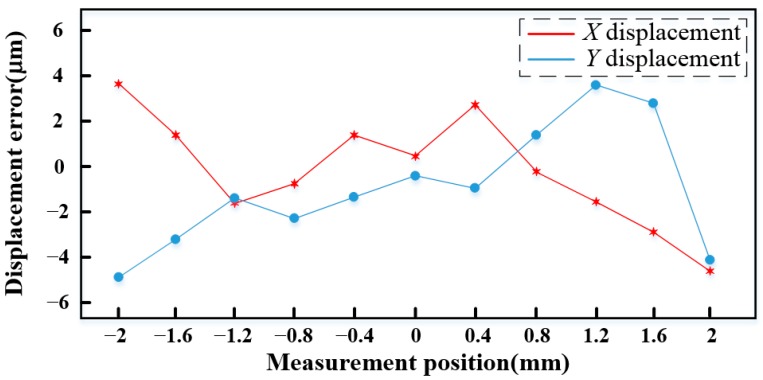
Measurement error of *XY* displacements.

The measurement error of the *XY* displacements is shown in Figure 18, the SS stage is controlled to move from −2 mm to 2 mm along the *X* direction and *Y* direction, and the errors at different positions are obtained by comparison of the data from the laser interferometers and the Hall sensors. It can be found that the measurement error of the *X* displacement is less than 4.6 μm, and that of the *Y* displacement is less than 4.8 μm in the whole stroke. 

**Figure 19:**
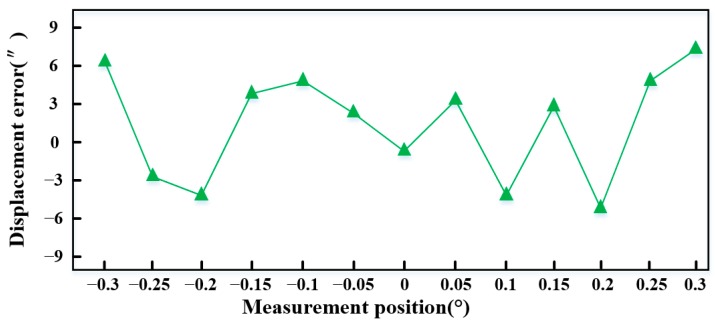
Measurement error of *θ_Z_* displacements.

The measurement error of the *θ_Z_* displacement is shown in Figure 19, the SS stage is controlled to rotate from −0.3° to 0.3° about the *Z* direction. It can be found that the measurement error of the *θ_Z_* displacement is less than 7.6″ in the whole stroke. 

## 6. Conclusions

In this paper, a three degrees of freedom displacement measurement system composed of three Hall sensors is presented. The measuring principle is analyzed, and the relationship between the variation of the position of the Hall sensors and the *XYθz* displacements of the SS stage is given. The output of one Hall sensor may correspond with many relative positions of the magnet and the sensor, but the combination of the output of three sensors can work out the only *XYθz* displacements of the SS stage. Therefore a magnetic field fitting method is proposed to build the relationship between the output of three sensors and the *XYθz* displacements, that is, the magnetic field of the magnets is a known quantity which is given by an elliptic function at a certain measurement height during displacement measurements. The parameters of the elliptic function are also fitted by analytic expression, so that the relative movement of the SS stage can be measured by the Hall sensors. The three degrees of freedom displacement measurement system is finally validated experimentally. 
